# Gonorrhea in the Female*Read at the East Texas Medico-Chirurgical Society, November, 1901.

**Published:** 1902-03

**Authors:** E. B. Parsons

**Affiliations:** Palestine, Texas


For Texas Medical Journal.

### Gonorrhea in the Female.*

BY E. B. PARSONS, M. D., PALESTINE, TEXAS.

By gonorrhea in the female we mean a specific inflammation of the genitalia of the female, produced by an invasion of the gonococcus of Neiser.

---

*Read at the East Texas Medico-Chirurgical Society, November, 1901.

The modes of invasion being too well known to mention here, we pass on to the more important description, and history of same.

The first symptom noticeable to a patient after impure intercourse are all the signs of inflammation of the vulvo vaginal outlet, with ardor urinæ, this rapidly producing a serous mucoserous and mucopurulent discharges, intense redness, heat and pain in the loin, and hypogastrium. With all these symptoms confronting us, we are pretty sure of a case of gonorrhea in the female.

If this is not sufficient evidence to form a diagnosis, we may subject secretion to microscopical inspection, where we will always find the gonococcus, if it be of a specific nature. In fact, the microscope will always clear up the diagnosis.

Were it not for the complications and sequelæ of gonorrhea in the female, our subject would not be worth your attention, for not until recently has this disease been looked upon in a serious light, as should have been, for not until recent years have gynecologists given it the attention it deserves; in all seriousness, gonorrhea is a serious disease, whether in the male or female; many a pure woman has gone to an untimely grave, many a pure life has been wrecked by pain, much less those that have been unsexed and rendered barren, deprived of that noble and God given power to reproduce the species, perhaps, too, infected by an innocent husband.

We do not have to go to the hovels, the houses of ill repute, always, to find the conditions just narrated, but we find splendid examples of them in high life. For this class of patients it seems to be more virulent, and it has a greater tendency to spread by continuity to the neighboring organs; for example, we may have following an acute vaginitis (specific) endometritis, metritis, cellulitis, peritonitis, salpingitis, ovaritis, pelvic abscess, cystitis, inflammation of the ureters, nephritis, endocarditis, abscess of vulva, vaginal glands.

*Treatment.*—We are continually in a state of transition in regard to treatment of gonorrhea; but viewed in the light of modern research, gonorrhea is due to a specific organism, the gonococcus of Neiser, and for this reason the old treatment of astringents and balsamics has yielded to a method which has for its chief aim the destruction of the gonococcus.

The great difficulty in this is due to the fact that the gonococci are not only found on the surface of the mucuos membrane, but in the deep layers as well, hence a remedy to be effective should be able to penetrate the deeper tissues without alteration by secretion, and also the constant use of disinfectants and germicides in "loco"

to render the habitation of the germs unsuitable for multiplication and extention.

For the former condition, to regulate the attack, I would recommend one of the recent silver salts, of which there are many, but my experience has been with protargol, say a 2 to 5 per cent solution, used by means of cotton swabs or introduced through a cylindrical speculum, the speculum withdrawn and the swab gradually drawn out; thus the entire vaginal surface is permeated with the solution. The labia should be kept separated by lint saturated in a mixture of bismuth and vasaline. For the latter condition named above, I would recommend frequent ablutions of hot water with permanganate of potash, bichloride of mercury, or any of the various antiseptics and disinfectants.

The ardor urinæ usually present can be relieved by demulcents diuretics and anodynes, of which bicarb. potash and tincture of hyoscyamus, spirits of nitre, cold water, or any of the numerous diuretic teas. This treatment, combined with antiphlogistic, regulating the bowels, hot sitz baths, with rest in the recumbent posture, will cure many cases of gonorrhea in from five to thirty days.

